# Desigualdades sociais no acesso aos alimentos: uma avaliação a partir do perfil de utilização dos locais de aquisição de alimentos no Município do Rio de Janeiro, Brasil

**DOI:** 10.1590/0102-311XPT159725

**Published:** 2026-07-20

**Authors:** Yoko Ametista Carvalho Suete Matos, Paulo César Pereira de Castro, Aline Alves Ferreira, Rosana Salles-Costa

**Affiliations:** 1 Universidade Federal do Rio de Janeiro, Rio de Janeiro, Brasil.

**Keywords:** Desigualdades Sociais, Ambiente Alimentar, Abastecimento de Alimentos, Social Inequalities, Food Social Space, Food Supply, Desigualdades Sociales, Espacio Social y Comida, Abastecimiento de Alimentos

## Abstract

O objetivo deste estudo foi avaliar a utilização dos locais de aquisição de alimentos e sua associação com desigualdades sociais, por meio de um estudo transversal baseado em amostra representativa de 1.855 domicílios no Rio de Janeiro, Brasil. A utilização dos locais de aquisição de alimentos foi avaliada por meio de um questionário e classificada segundo o seu tipo de venda. O perfil social foi classificado com base nas informações dos chefes das famílias. Empregou-se o modelo de regressão logística para avaliar a associação entre a escolaridade e os locais de aquisição de alimentos. Mais de 2/3 das famílias citaram os supermercados (97,5%) e locais de venda de alimentos *in natura* ou minimamente processados (80,6%) como locais de compra. A menor escolaridade do chefe da família (OR = 2,1; IC95%: 1,3-3,4) e a ausência de trabalho remunerado (OR = 1,38; IC95%: 1,24-2,28) se relacionaram significativamente a maior aquisição em locais de venda de alimentos *in natura* ou minimamente processados. Como fatores de proteção, os níveis severos de insegurança alimentar (OR = 0,3; IC95%: 0,2-0,7), a ausência de trabalho remunerado (OR = 0,6; IC95%: 0,5-0,8) e a escolaridade dos chefes das famílias (5 a 11 anos; OR = 0,55; IC95%: 0,33-0,93) foram respectivamente associados a menor compra de alimentos em supermercados, pequenos mercados e em locais de venda de produtos ultraprocessados. A modificação do ambiente alimentar favorecerá a garantia do direito humano à alimentação adequada por meio do acesso a alimentos saudáveis, dado que sua realização pressupõe um abastecimento equitativo e sustentável para a população.

## Abstract (en)

This study aimed to evaluate the use of food purchase locations and their association with social inequalities. This cross-sectional study was based on a representative sample of 1,855 households in Rio de Janeiro, Brazil. The use of food purchase locations was assessed using a questionnaire and classified according to the type of food retail outlet. Social profile was defined based on information about the head of household. Logistic regression models were used to evaluate the association between educational attainment and types of food purchase locations. Over two-thirds of households reported supermarkets (97.5%) and outlets selling fresh or minimally processed foods (80.6%) as their main food purchasing establishments. Lower educational attainment of the head of household (OR = 2.1; 95%CI: 1.3-3.4) and lack of paid employment (OR = 1.38; 95%CI: 1.24-2.28) were significantly associated with greater purchasing from outlets selling fresh or minimally processed foods. As protective factors, severe levels of food insecurity (OR = 0.3; 95%CI: 0.2-0.7), lack of paid employment (OR = 0.60; 95%CI: 0.5-08), and the educational attainment of the head of household (5-11 years; OR = 0.55; 95%CI: 0.33-0.93) were associated with lower purchasing at supermarkets, small grocery stores, and outlets selling ultra-processed foods, respectively. Changes in the food environment may help ensure the human right to adequate food by improving access to healthy foods, as its realization depends on equitable and sustainable food supply systems for the population.

## Resumen

El objetivo de este estudio fue evaluar el uso de los lugares de adquisición de alimentos y su asociación con las desigualdades sociales. Se trata de un estudio transversal basado en una muestra representativa de 1.855 hogares en Rio de Janeiro, Brasil. El uso de los lugares de adquisición de alimentos se evaluó mediante un cuestionario y se clasificó según el tipo de venta de alimentos. El perfil social se clasificó según la información proporcionada por los jefes de familia. Se realizó un modelo de regresión logística para evaluar la asociación entre el nivel educativo y los lugares de adquisición de alimentos. Más de dos tercios de las familias mencionaron los supermercados (97,5%) y los lugares de venta de alimentos frescos o mínimamente procesados (80,6%) como lugares de compra de alimentos. El menor nivel educativo del jefe de familia (OR = 2,1; IC95%: 1,3-3,4) y la ausencia de trabajo remunerado (OR = 1,38; IC95%: 1,24-2,28) se asociaron significativamente con una mayor adquisición en lugares de venta de alimentos frescos o mínimamente procesados. Como factores de protección, los niveles graves de inseguridad alimentaria (OR = 0,3; IC95%: 0,2-0,7), la ausencia de trabajo remunerado (0,60; IC95%: 0,5-08) y la escolaridad del jefe de familia (5 a 11 años; OR = 0,55; IC95%: 0,33-0,93) se asociaron respectivamente con una menor compra de alimentos en supermercados, pequeños mercados y lugares de venta de productos ultraprocesados. La modificación del entorno alimentario favorecerá la garantía del derecho humano a una alimentación adecuada, mediante el acceso a alimentos saludables, dado que su realización pasa por un abastecimiento equitativo y sostenible para la población.

## Introdução

O ambiente alimentar é definido por oportunidades e condições presentes nos ambientes físico, econômico, político e sociocultural, que influenciam as escolhas alimentares e o estado nutricional dos indivíduos ^1^. Esse ambiente também é influenciado por determinantes individuais que impactam no consumo alimentar, como a renda, a escolaridade, o preço, as habilidades culinárias, e por normas políticas, sociais e culturais que moldam o comportamento alimentar do indivíduo ^2^. Sob essa perspectiva, o ambiente alimentar tem sido apontado como espaço de interação do consumidor com o sistema alimentar, relacionando-se com a complexa rede de componentes de abastecimento.

No âmbito das classificações do ambiente alimentar, a de varejo define-se como o local em que os alimentos estão disponíveis, envolvendo a quantidade, o tipo, a localização e o acesso aos estabelecimentos que comercializam os alimentos ^3^. Nesse contexto, fatores socioeconômicos são determinantes para o acesso à alimentação ^4^ ^,^ ^5^. Questões raciais ^6^ ^,^ ^7^ ^,^ ^8^ ^,^ ^9^, segregação econômica ^4^ ^,^ ^10^, menor nível de escolaridade ^11^ e níveis baixos de renda ^12^ ^,^ ^13^ ^,^ ^14^ ^,^ ^15^ são alguns desses elementos que comprometem o acesso ao ambiente alimentar.

No Brasil, estudos têm apresentado o impacto das desigualdades socioeconômicas e demográficas nessa dinâmica ^16^ ^,^ ^17^ ^,^ ^18^, de modo que a distribuição dos estabelecimentos não ocorre de forma igualitária nas unidades territoriais ^11^ ^,^ ^19^. Nas grandes metrópoles, como São Paulo e Rio de Janeiro, observa-se que bairros ou entornos de espaços escolares com menor renda e piores indicadores socioeconômicos são os mesmos locais com maior presença de desertos e pântanos alimentares apresentando baixa disponibilidade de locais de vendas de alimentos in natura ou minimamente processados ^20^ ^,^ ^21^.

Além disso, o local de moradia interfere também no tipo de alimentos adquiridos pela população ^13^. Nesse cenário, o uso de supermercados é predominante, especialmente nas áreas urbanas ^13^, funcionando como fontes de alimentos *in natura* ou minimamente processados e ultraprocessados ^22^. Observa-se que, com o aumento da renda e do poder aquisitivo, há uma tendência à maior aquisição em supermercados, com destaque para os alimentos ultraprocessados ^13^.

Porém, ainda que a disponibilidade de alimentos seja um indicador relevante das condições de acesso, ela não abrange todas as dimensões dessa questão. Tradicionalmente, os estudos que avaliam esse contexto investigam a distribuição dos estabelecimentos no território, mas não avaliam a interação dos consumidores com o ambiente alimentar de varejo, especialmente em grandes centros urbanos. Sendo assim, o avanço deste estudo reside na incorporação da perspectiva do consumidor sobre o ambiente de varejo. Ao focar na utilização e na interação dos indivíduos com os locais de aquisição, e não apenas na distribuição geográfica dos estabelecimentos, é possível identificar desigualdades sociais que não são capturadas por macroindicadores de disponibilidade. Portanto, este estudo foi conduzido com o objetivo de avaliar a utilização dos locais de aquisição de alimentos no Município do Rio de Janeiro, identificando desigualdades sociais no acesso.

## Método

### Desenho do estudo e coleta de dados

Trata-se de um estudo transversal que utilizou a base de dados do *I Inquérito Populacional sobre a Insegurança Alimentar no Município do Rio de Janeiro*, realizado no período de novembro de 2023 a janeiro de 2024, com o objetivo de avaliar os níveis de insegurança alimentar da população carioca ^23^.

A amostra é representativa de 2 mil domicílios da área urbana do município e foi planejada considerando uma margem de erro de 4,9% para estimativas pontuais, seus intervalos de 95% de confiança (IC95%), contemplando as cinco Áreas de Planejamento (AP) e com 400 domicílios cada. Esta margem foi adequada para estimar o nível de insegurança alimentar grave na ordem de até 15%, com base no inquérito populacional realizado no ano de 2022 ^24^. As entrevistas foram distribuídas proporcionalmente pelas Regiões Administrativas e bairros que compõem cada AP, utilizando um delineamento por conglomerados, em três estágios de seleção dos domicílios: sorteio dos setores censitários dos bairros de cada Região Administrativa; seleção das ruas nos respectivos setores censitários e seleção de domicílios entrevistados. Maiores detalhes sobre o desenho amostral estão disponíveis no relatório final do estudo ^23^.

Entrevistadores treinados pela equipe de pesquisadores realizaram entrevistas face a face com a pessoa de referência da família, ou um adulto capaz de informar com precisão as características demográficas de todos os moradores do domicílio, bem como dados relativos à alimentação, à renda familiar e ao peso e à altura de todos os moradores.

Para as entrevistas, foi desenvolvido um aplicativo para aparelhos celulares contendo 80 perguntas elaboradas com base em inquéritos anteriores ^24^ ^,^ ^25^, organizado nos seguintes módulos: (1) identificação do domicílio e da família; (2) perfil familiar; (3) renda dos últimos 30 dias; (4) *Escala Brasileira de Insegurança Alimentar* (EBIA); (5) *Escala da Insegurança Hídrica*; (6) apoio social e questões que envolvem a alimentação; (7) informações sobre o local de moradia (percepção da vizinhança); (8) local de aquisição de alimentos; e, (9) estado nutricional. Neste estudo, as perguntas dos módulos 1, 2, 3, 4 e 8 foram avaliadas. A cópia do questionário encontra-se disponível em publicação anterior ^23^.

### Locais para a aquisição de alimentos

Para a classificação dos locais de aquisição de alimentos, foram consideradas as informações sobre o principal ponto de compra dos seguintes itens: arroz, feijão, legumes e verduras, frutas, carnes, ovos, leite e derivados, doces, biscoitos e salgadinhos e bebidas açucaradas. Essa escolha deve-se ao fato de serem marcadores de uma alimentação saudável ou não saudável, com base em estudos anteriores ^26^ ^,^ ^27^ e nos componentes avaliados no módulo sobre alimentação do Sistema de Vigilância Alimentar e Nutricional (SISVAN) ^28^.

Para os locais de aquisição, o questionário considerou as seguintes opções: supermercados/hipermercados, atacadistas, pequenos mercados, açougues e peixarias, quitandas, hortifrutis ou sacolões, feiras livres, padarias ou outros locais. Tais categorias foram definidas a partir da adaptação dos exemplos utilizados na aquisição coletiva da *Pesquisa de Orçamentos Familiares* (POF) de 2017 e 2018 ^29^. As categorias “aquisição informal” (n = 198 [10,7%]) e “doações” não foram incluídas na análise multivariada devido à baixa frequência de respostas (n = 261 [14,1%]). Apesar de desempenharem papel central em contextos de insegurança alimentar, configurando-se como estratégias complementares de acesso a alimentos, a reduzida representatividade dessas categorias na amostra geraria estimativas enviesadas nos modelos de regressão logística. O Quadro 1 apresenta o detalhamento da organização dos locais de aquisição ^23^.

**Quadro 1:** Locais de aquisição e detalhamento segundo características específicas de comercialização.

LOCAL DE AQUISIÇÃO	DETALHAMENTO SEGUNDO AS CARACTERÍSTICAS DOS DIVERSOS LOCAIS
Supermercados	Supermercados e atacadistas
Pequenos mercados	Mercearia, minimercados ou armazém; padarias
Locais de venda predominantemente de alimentos *in natura* e/ou minimamente processados	Feira; açougues/peixarias; quitanda, hortifruti/sacolão; aviário; central de abastecimento
Locais de venda predominantemente de produtos ultraprocessados	Lojas especializadas em balas e doces; conveniência; casa de venda de biscoitos; lojas de venda de chocolates; depósito de bebidas; bar; farmácia
Informal	Vendinhas; venda porta a porta/vendedor; caminhão ou carro que passava na rua; camelô
Doações de alimentos	Cestas básicas; doações

### Demais variáveis de estudo

Com relação ao perfil das famílias, foram avaliadas informações sobre: renda familiar *per capita*, em salários mínimos (< 1/2, 1/2-1, > 1); perfil da pessoa de referência do domicílio (sexo [homem/mulher]; raça/cor da pele por autoidentificação, conforme as categorias estabelecidas pelo Instituto Brasileiro de Geografia e Estatística (IBGE) [branco, preto, pardo], excluindo as famílias autoidentificadas como indígenas e amarelas pela não representatividade da amostra e baixo percentual [indígenas: n = 13; 0,62%; amarelos: n = 10; 0,45%] ^30^; escolaridade, em anos [≤ 4, 5-11, ≥ 12] e a presença ou não de trabalho remunerado [sim, não]). Adicionalmente, foi utilizado o indicador de segurança alimentar avaliado pela EBIA para avaliar o nível de segurança alimentar e os graus de insegurança alimentar das famílias (leve, moderada e grave) ^31^.

A localização dos domicílios foi avaliada de acordo com sua distribuição nas cinco APs do município: AP1 - Centro e Zona Portuária; AP2 - Zona Sul e Tijuca; AP3 - Zona Norte; AP4 - Barra da Tijuca, Jacarepaguá e Cidade de Deus; AP5 - outros bairros da Zona Oeste.

### Análises estatísticas

Foram realizadas análises descritivas com estimativas percentuais e IC95% para caracterizar a população estudada e a prevalência da utilização dos locais de aquisição de alimentos. Em seguida, procedeu-se à análise estratificada das características socioeconômicas avaliadas segundo esses locais.

Ademais, foram desenvolvidos modelos de regressão logística para estimar os *odds ratios* (OR) e seus respectivos IC95%, a fim de testar a associação entre as variáveis socioeconômicas e a utilização dos locais de aquisição de alimentos, separadamente. Para o ajuste, consideraram-se as variáveis que se associaram ao nível de até 20% na análise estratificada. Os modelos foram ajustados pelas APs, com significância de 5%. As análises foram realizadas no programa estatístico Stata 16.0 (https://www.stata.com), utilizando o pacote *svy* para as estimativas e para incorporar o desenho amostral do estudo.

O inquérito foi aprovado pelo Comitê de Ética em Pesquisa do Hospital Universitário Clementino Fraga Filho, Universidade Federal do Rio de Janeiro, em 2021 (CAAE: 54473421.6.0000.5257), sendo conduzido de acordo com todas as diretrizes éticas para estudos com seres humanos no país.

## Resultados

**Figura 1:**
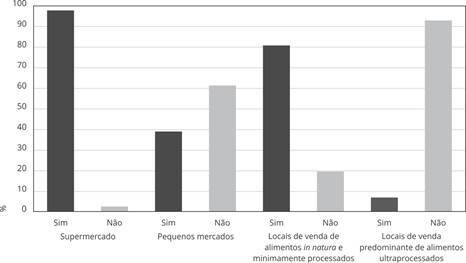
Distribuição percentual da utilização dos estabelecimentos para a aquisição de alimentos por chefes de domicílio no Município do Rio de Janeiro, Brasil.

Dos domicílios avaliados, 1.855 (92,7%) compuseram a amostra deste estudo. O perfil sociodemográfico das famílias caracterizou-se pelo predomínio de chefes do sexo feminino, pardos, com escolaridade intermediária e inserção no mercado de trabalho remunerado. Observou-se também maior proporção de famílias em segurança alimentar e com renda *per capita* > 1 salário mínimo (Tabela 1). Os supermercados figuraram como os principais locais de compra, seguidos pelos estabelecimentos que comercializam alimentos *in natura* ou minimamente processados e pelos pequenos mercados. Pontos de venda de produtos ultraprocessados foram os menos utilizados como locais de aquisição (Figura 1).

**Tabela 1:** Distribuição percentual e respectivos intervalos de 95% de confiança (IC95%) do perfil das famílias no Município do Rio de Janeiro, Brasil, 2024.

Variáveis sociodemográficas	% (IC95%)
Perfil do chefe da família	
Sexo	
Masculino	46,7 (44,0-49,3)
Feminino	53,4 (50,7-56,0)
Raça/Cor	
Branca	28,2 (25,9-30,6)
Preta	26,6 (24,3-29,0)
Parda	45,2 (42,6-47,8)
Escolaridade (anos)	
≤ 4	15,2 (13,4-17,2)
5-11	62,7 (60,1-65,1)
≥ 12	22,1 (20,1-24,3)
Trabalho remunerado	
Sim	59,1 (56,5-61,7)
Não	40,9 (38,3-43,5)
Características dos domicílios	
Renda familiar *per capita* (salários mínimos)	
Até 1/2	21,6 (19,3-24,0)
1/2 a 1	29,8 (27,3-32,5)
Mais de 1	48,6 (45,8-51,4)
Segurança e níveis de insegurança alimentar *	
Segurança alimentar	66,7 (64,1-69,2)
Insegurança alimentar leve	17,1 (15,1-19,2)
Insegurança alimentar moderada e grave	16,3 (14,3-18,4)
Áreas de Planejamento	
AP1	4,7 (4,2-5,2)
AP2	16,1 (14,6-17,8)
AP3	38,1 (35,4-40,9)
AP4	14,6 (13,2-16,1)
AP5	26,5 (24,3-28,9)

* Avaliada pela *Escala Brasileira de Insegurança Alimentar* considerando os níveis de severidade da insegurança alimentar na mesma categoria.

Quando estratificada por variáveis sociodemográficas, a utilização dos locais de aquisição de alimentos revelou padrões distintos. Famílias em que o chefe tinha a menor escolaridade apresentaram significativamente menor dependência de supermercados e maior frequência de compra em estabelecimentos de alimentos *in natura* ou minimamente processados. A presença de atividade remunerada do responsável pelo domicílio relacionou-se à maior utilização de pequenos mercados e de locais de ultraprocessados (valor de p < 0,05), enquanto o aumento da renda familiar *per capita* indicou um gradiente de maior utilização de pontos de venda com predominância de ultraprocessados (valor de p < 0,05).

Quanto ao indicador de segurança e insegurança alimentar, destaca-se que o maior percentual de famílias em segurança alimentar optou por estabelecimentos de venda de ultraprocessados (valor de p < 0,05). As desigualdades territoriais também foram evidentes, especialmente na Zona Oeste, onde houve maior utilização de pequenos mercados e locais de venda de alimentos *in natura*, indicando especificidades no acesso segundo o território de residência (Tabela 2).

**Tabela 2:** Distribuição percentual e respectivos intervalos de 95% de confiança (IC95%) dos indicadores sociodemográficos segundo o perfil de utilização dos locais de aquisição de alimentos. Município do Rio de Janeiro, Brasil, 2024.

Indicadores sociodemográficos	Supermercado		Pequeno mercado		**Venda de alimentos *in natura* e minimamente processados**		Venda predominante de produtos ultraprocessados	
	% (IC95%)	Valor de p *	% (IC95%)	Valor de p *	% (IC95%)	Valor de p *	% (IC95%)	Valor de p *
Perfil do chefe da família								
Sexo								
Masculino	97,1 (95,7-98,1)	0,44	41,5 (37,8-45,4)	0,06	78,8 (75,4-81,7)	0,14	7,0 (5,4-9,0)	0,92
Feminino	97,7 (96,4-98,6)		36,5 (33,1-40,1)		81,9 (79,0-84,6)		6,9 (5,4-8,7)	
Raça/Cor		0,09		< 0,05		0,22		0,07
Branca	97,5 (95,5-98,6)		33,9 (29,4-38,7)		80,6 (76,4,84,1)		9,3(6,9-12,3)	
Preta	96,1 (93,6-97,7)		44,8 (39,8-49,9)		83,6 (79,4-87,1)		5,3 (3,5-8,0)	
Parda	98,3 (97,0-99,0)		38,2 (34,4-42,1)		79,1 (75,7-82,2)		6,9 (5,3-8,9)	
Escolaridade (anos)		< 0,05		< 0,001		< 0,05		< 0,001
≤ 4	93,4 (89,6-95,8)		35,2 (29,1-41,8)		85,2 (79,5-89,4)		3,6 (1,8-7,3)	
5-11	98,2 (97,1-98,9)		42,9 (39,6-46,3)		81,5 (78,7-84,0)		5,6 (4,4-7,3)	
≥ 12	98,2 (95,8-99,3)		29,4 (24,9-70,6)		75,0 (70,2-79,3)		13,6 (10,6-17,3)	
Trabalho remunerado		0,23		< 0,001		< 0,05		< 0,05
Sim	97,9 (96,8-98,6)		43,3 (40,0-46,6)		77,5 (74,6-80,2)		8,3 (6,8-10,1)	
Não	96,9 (95,1-98,1)		32,2 (28,4-36,3)		85,1 (81,8-87,9)		5,4 (3,8-7,5)	
Características dos domicílios								
Renda familiar *per capita* (salários mínimos)		0,23		0,17		0,38		< 0,05
Até 1/2	97,6 (94,8-98,9)		44,0 (37,9-50,4)		80,3 (74,7-84,8)		3,9 (2,1-6,9)	
1/2 a 1	96,2 (94,1-97,6)		41,9 (36,8-47,1)		83,1 (78,8-86,7)		5,2 (3,4-7,8)	
Mais de 1	97,9 (96,5-98,8)		37,7 (34,0-41,5)		79,4 (76,0-82,4)		7,8 (6,1-10,0)	
Segurança e níveis de insegurança alimentar **		< 0,05		0,41		0,89		< 0,05
Segurança alimentar	98,1 (97,1-98,8)		37,4 (34,4-40,5)		80,9 (78,3-83,3)		9,1 (7,6-10,9)	
Insegurança alimentar leve	97,8 (95,2-99,0)		40,4 (34,1-47,0)		79,6 (73,8-84,4)		3,0 (1,5-6,2)	
Insegurança alimentar moderada e grave	94,5 (90,6-96,9)		42,3 (35,6-49,2)		80,8 (74,6-85,7)		3,3 (1,6-6,9)	
Áreas de Planejamento (AP)		< 0,05		< 0,001		< 0,001		< 0,001
AP1	96,2 (93,7-97,7)		38,3 (33,5-43,4)		76,6 (72,0-80,7)		9,0 (6,4-12,4)	
AP2	99,2 (97,5-99,7)		26,6 (22,4-31,4)		81,6 (77,4-85,2)		13,4 (10,3-17,2)	
AP3	98,1 (96,1-99,1)		35,5 (30,8-40,5)		76,3 (71,7-80,3)		3,2 (1,8-5,5)	
AP4	94,2 (91,3-96,1)		41,2 (36,3-46,3)		76,3 (71,8-80,4)		8,9 (6,4-12,2)	
AP5	97,6 (95,4-98,7)		49,5 (44,4-54,7)		89,3 (85,6-92,1)		7,7 (5,3-10,9)	

* Qui-quadrado;

** Avaliada pela *Escala Brasileira de Insegurança Alimentar* considerando os níveis de severidade da insegurança alimentar na mesma categoria.

Nos modelos multivariados (Tabela 3), permaneceram associações importantes após os ajustes. Em relação aos supermercados, famílias em insegurança alimentar moderada/grave apresentaram menor chance de utilizar esses locais em comparação àquelas em segurança alimentar (valor de p < 0,001). Quanto aos pequenos mercados, a análise do perfil das famílias indicou que chefes pretos ou com escolaridade entre 5 e 11 anos apresentaram maiores chances de utilização, enquanto a ausência de trabalho remunerado reduzia em 40% essa probabilidade (valor de p < 0,001). Sobre os estabelecimentos de alimentos *in natura* e minimamente processados, possuir menor escolaridade e não possuir trabalho remunerado aumentaram significativamente a probabilidade de escolha desses estabelecimentos. Já os locais de venda de ultraprocessados associaram-se inversamente à escolaridade entre 5 e 11 anos o que reduzia a chance de as famílias optarem por estes locais (valor de p < 0,05).

**Tabela 3:** Razão de chances ajustada (OR) e seus intervalos de 95% de confiança (IC95%) da relação entre indicadores socioeconômicos e o perfil de utilização dos locais de aquisição de alimentos do Município do Rio de Janeiro, Brasil, 2024.

Indicadores socioeconômicos	Supermercados	Pequenos mercados	**Locais de venda de alimentos *in natura* ou minimamente processados**	Locais de venda de produtos ultraprocessados
OR (IC95%)	OR (IC95%)	OR (IC95%)	OR (IC95%)
Perfil do chefe da família				
Sexo				
Masculino Feminino	-	1,0	1,0	-
Feminino	-	0,9 (0,7-1,1)	1,1 (0,9-1,5)	-
Raça/Cor				
Branca	1,0	1,0	-	1,0
Preta	0,9 (0,4-2,0)	1,5 (1,1-2,0) *	-	0,7 (0,4-1,4)
Parda	1,8 (0,8-4,2)	1,1 (0,8-1,4)	-	0,8 (0,5-1,4)
Escolaridade (anos)				
≤ 4	0,3 (0,1-1,1)	1,4 (0,9-2,1)	1,7 (1,1-2,8) *	0,5 (0,2-1,2)
5-11	1,0 (0,3-3,2)	1,8 (1,4-2,4) **	1,5 (1,1-2,0) *	0,6 (0,3-0,9) *
≥ 12	1,0	1,0	1,0	1,0
Trabalho remunerado				
Sim	-	1,0	1,0	1,0
Não	-	0,6 (0,5-0,7) **	1,7 (1,2-2,3) **	0,9 (0,5-1,5)
Características dos domicílios				
Renda familiar *per capita* (salários mínimos)				
Até 1/2	-	-	-	1,1 (0,5-2,3)
1/2 a 1	-	-	-	1,1 (0,6-2,0)
Mais de 1	-	-	-	1,0
Segurança e níveis de insegurança alimentar ***				
Segurança alimentar	1,0	-	1,0	1,0
Insegurança alimentar leve	0,8 (0,3-2,2)	-	0,9 (0,6-1,3)	0,5 (0,2-1,2)
Insegurança alimentar moderada e grave	0,3 (0,2-0,7) *	-	0,8 (0,5-1,3)	0,6 (0,3-1,4)

* Valor de p < 0,05;

** Valor de p < 0,001;

*** Avaliada pela *Escala Brasileira de Insegurança Alimentar* considerando os níveis de severidade da insegurança alimentar na mesma categoria.

## Discussão

Assim como em outras grandes metrópoles brasileiras, o Município do Rio de Janeiro é marcado por profundas e históricas desigualdades sociais e territoriais. A menor escolaridade dos chefes das famílias revela-se um importante determinante dessas condições, estando associada à menor compra de alimentos em locais de venda de ultraprocessados e à maior frequência naqueles que vendiam majoritariamente itens *in natura* e minimamente processados. Esses mesmos locais foram mais utilizados por chefes sem remuneração, o que evidencia a relevância desses estabelecimentos para a população em situação de maior vulnerabilidade.

Outrossim, é importante destacar que, sendo o Rio de Janeiro uma das mais importantes metrópoles do país, tanto do ponto de vista sociocultural quanto econômico, a aquisição de alimentos foi majoritariamente feita nos supermercados. Nesse sentido, há indícios de uma uniformização no comércio varejista do município, padrão alinhado a outros achados em estudos populacionais conduzidos no Brasil, como o realizado a partir de dados da POF 2017-2018 ^32^.

Apesar disso, as compras nesses locais se associaram às condições sociodemográficas mais favoráveis das famílias cariocas (maior escolaridade do chefe das famílias e presença de segurança alimentar). É possível que essas famílias tendam a utilizar mais os supermercados por disporem de maior capacidade de consumo, acesso mais estável ao transporte e a práticas de compras planejadas, além de aderirem com maior frequência ao padrão de consumo dominante nas grandes cidades, que privilegia a conveniência, a variedade e a percepção qualidade e segurança dos produtos ^4^ ^,^ ^13^.

As grandes redes de supermercados ingressaram no Brasil durante as décadas de 1980 e 1990, período marcado por uma abertura econômica concomitante com uma ausência de regulação estatal no abastecimento alimentar das cidades. Tal cenário levou a uma ramificação dessas redes no país, com uma expansão do domínio ao longo dos anos, especialmente em grandes centros urbanos como o Rio de Janeiro ^33^. Sabe-se que esses estabelecimentos são locais de venda tanto de produtos ultraprocessados como de alimentos *in natura*. Porém, apesar dessa característica mista, eles tendem a influenciar o maior consumo de ultraprocessados dada a maior oferta e as estratégias mercadológicas utilizadas para incentivar sua venda ^32^ ^,^ ^34^ ^,^ ^35^ ^,^ ^36^.

Associado a isso, os alimentos ultraprocessados tornaram-se mais baratos que os alimentos *in natura* ou minimamente processados, antecipando a previsão de que tal fenômeno ocorreria apenas em 2026 ^37^, seguindo a tendência encontrada na América Latina ^38^. Desta maneira, tais características sugerem que esse tipo de estabelecimento apresenta baixa saudabilidade, facilitando o consumo de itens de pior qualidade nutricional.

É importante destacar que a expansão dos supermercados foi acompanhada por profundas transformações no ambiente alimentar, resultando na redução de estabelecimentos especializados na venda de alimentos *in natura*, como hortifrutigranjeiros, açougues e peixarias, além do enfraquecimento do pequeno varejo tradicional, isto é, mercearias, armazéns e mercados de bairro ^39^. Esse fenômeno ocorreu, em grande parte, porque os supermercados absorveram setores antes independentes, consolidando-se como os principais pontos de abastecimento. Além disso, o alto poder de negociação e controle sobre os preços exercido por esses estabelecimentos impacta negativamente pequenos produtores e comerciantes locais, dificultando sua manutenção no mercado de modo competitivo ^35^ ^,^ ^40^.

No contexto dos determinantes comerciais da saúde - definidos como os sistemas, práticas e caminhos pelos quais os atores comerciais impulsionam a saúde e a equidade ^32^ - os supermercados exercem um papel central no favorecimento de um ambiente alimentar que afeta negativamente a saúde da população. O predomínio desses estabelecimentos no abastecimento de diversos estados brasileiros ^32^ promove uma ampla oferta de produtos ultraprocessados, vendidos a preços cada vez mais baixos e com fortes estratégias de marketing ^35^ ^,^ ^36^. Dessa forma, os supermercados possuem uma influência significativa na formação dos hábitos alimentares da população, incentivando tanto o consumo de alimentos *in natura* como também de produtos não saudáveis, como os ultraprocessados ^41^.

Apesar disso, em algumas regiões do Município do Rio de Janeiro (Zona Oeste e Zona Norte), equipamentos de venda de alimentos como feiras livres, sacolões, quitandas, açougues e peixarias são exemplos de modelos que resistem ao tempo e ao avanço supermercadista. Esses locais são importantes para uma oferta diversificada de alimentos, especialmente aqueles *in natura* ou minimamente processados, que, segundo o *Guia Alimentar para a População Brasileira* ^42^, devem constituir a base da alimentação. Ademais, são espaços de convivência, nos quais se estreitam laços entre os usuários, comerciantes e produtores da comunidade ^43^, exercendo papel fundamental na circulação de recursos, na provisão de novos postos de trabalho, na geração de renda e no fortalecimento do circuito da economia local ^44^.

Nesse sentido, Silva et al. ^22^ propuseram uma nova classificação dos locais de aquisição seguindo a classificação NOVA, apontando que os hortifrutigranjeiros, as peixarias, os açougues e os restaurantes foram identificados, em todas as regiões do país, como voltados exclusivamente para a venda de alimentos *in natura*, minimamente processados e de ingredientes culinários. Essa classificação corrobora a importância desses locais como componentes estratégicos na construção de sistemas alimentares mais saudáveis e sustentáveis.

Contudo, ao considerar os fatores sociodemográficos que influenciam no uso desses estabelecimentos, a escolaridade dos responsáveis pela família se mostrou um marcador importante em relação ao tipo de estabelecimento utilizado no ambiente alimentar do Município do Rio de Janeiro. As menores faixas de escolaridade aumentaram a chance de compra tanto em locais de aquisição de alimentos *in natura* e minimamente processados como em pequenos mercados, diminuindo a possibilidade em relação aos locais de venda de produtos ultraprocessados. Esses dados sugerem que o acesso a maiores níveis de escolaridade, neste contexto específico, não se traduz necessariamente em uma maior adesão a locais considerados mais saudáveis para a aquisição de alimentos.

Quando o chefe familiar não possuía trabalho remunerado, houve maior associação à compra em locais de venda de alimentos *in natura* ou minimamente processados. Adicionalmente, a insegurança alimentar moderada e grave foi um fator de proteção para a menor aquisição em supermercados, sendo outro resultado importante que retrata que estabelecimentos como os pequenos mercados e pontos de venda de alimentos *in natura* e minimamente processados costumam ser mais próximos à residência das famílias, proporcionando maior praticidade para o dia a dia ^5^, o que indica a importância da localização e do território. Entretanto, a literatura indica que os pequenos mercados tendem a impor valores mais altos nos alimentos comercializados, além de apresentarem baixa oferta de alimentos saudáveis ^22^ ^,^ ^40^ e a grande oferta de alimentos ultraprocessados ^45^, o que leva ao maior comprometimento da renda familiar, ocasionando uma diminuição na quantidade ou na qualidade dos produtos adquiridos.

O perfil de utilização dos locais de aquisição de alimentos pode ainda refletir desigualdades estruturais atravessadas por marcadores de raça/cor da pele. No caso de famílias chefiadas por pessoas autodeclaradas pretas, o padrão de acesso a determinados estabelecimentos pode ser efeito de um processo histórico de marginalização da população negra no Brasil, marcado pela escravização e por uma abolição desacompanhada de reparações estatais ou políticas públicas eficazes de inserção no mercado de trabalho e de acesso à terra e ao território urbano ^46^. No município estudado, esse processo contribuiu para o deslocamento dessa população para áreas mais empobrecidas da cidade ou para a formação de favelas ^47^, territórios nos quais os pequenos mercados são frequentemente encontrados ^48^. Resultado semelhante foi identificado em estudo realizado no Sul do Brasil, que evidenciou a associação positiva entre a autodeclaração de raça/cor da pele negra e a exposição a ambientes alimentares menos saudáveis ^6^.

Outro achado relevante foi observado em famílias residentes na Zona Oeste (AP5) do município, dado que quase 50% utilizaram os pequenos mercados para compra de alimentos. Essa região, caracterizada por um grande contingente populacional e vasta extensão territorial, é marcada por desigualdades econômicas e sociais e pela violência associada a grupos armados que exercem poder paralelo ao Estado ^49^. A violência cotidiana vivenciada por populações semelhantes à avaliada neste estudo pode contribuir para a preferência por pequenos mercados, uma vez que reduzem o deslocamento geográfico e, consequentemente, diminuem o risco de exposição a conflitos. Além disso, a própria violência dificulta a implementação de outros tipos de comércios ^50^ ^,^ ^51^, limitando as opções disponíveis aos moradores, principalmente no que se refere ao acesso a alimentos saudáveis. 

Neste viés, pode-se recorrer à teoria dos circuitos urbanos de Milton Santos ^52^, segundo a qual a economia urbana é formada por dois circuitos distintos e interdependentes: o superior e o inferior. O circuito superior é constituído por estabelecimentos como bancos, comércios, indústrias e serviços modernos, transportadoras e atacadistas, refletindo os avanços tecnológicos ocorridos no território. Já o circuito inferior é caracterizado por comércios e serviços menos estruturados, normalmente voltados para a população em situação de vulnerabilidade. Sob a perspectiva dessa teoria, a Zona Oeste do Rio de Janeiro (AP5), área historicamente marcada pela desigualdade social e territorial, insere-se no circuito inferior, o que explica parte dos resultados do presente estudo. Ressalta-se que essa diferenciação entre circuitos provém não só das desigualdades na formação das cidades, mas também dos interesses dos agentes políticos e comerciais ^52^.

A utilização dos locais de venda de ultraprocessados esteve associada com os marcadores de melhor condição de vida, como a maior faixa de renda e de escolaridade, além da presença de segurança alimentar. Os achados desta pesquisa corroboram com estudos recentes que apresentam a associação do consumo desses alimentos a situações socioeconômicas mais favoráveis ^53^ ^,^ ^54^.

Porém, observa-se no Brasil uma mudança de tendência nesse padrão de consumo, possivelmente devido ao barateamento desses produtos, ao aumento de preço de alimentos frescos ^37^, à ampliação das compras em supermercados, à utilização de estratégias de marketing diversificadas, bem como ao aumento do poderio econômico de empresas transnacionais ^54^.

Os resultados deste estudo apresentam evidências que corroboram com as propostas do Governo Federal brasileiro e que dialogam com o tema de ambientes alimentares, vigentes tanto no plano nacional de abastecimento alimentar como no de segurança alimentar e nutricional. Dentre seus diversos objetivos, está a criação de uma rede de varejo saudável, com foco em locais de vulnerabilidade ou com desertos e pântanos alimentares ^55^. Desta forma, os pequenos mercados podem ser utilizados nessa estratégia, uma vez que já estão localizados próximos às residências, especialmente em bairros mais vulneráveis em relação à estrutura urbana e social. Para tanto, será necessário apoiar os pequenos comerciantes para que haja uma modificação no perfil dos produtos ofertados nesses estabelecimentos, considerando que atualmente esses disponibilizam mais alimentos ultraprocessados e/ou praticam preços menos atraentes para alimentos *in natura* e minimamente processados ^56^.

A ampliação de equipamentos de comércio que priorizam a venda de alimentos *in natura*, bem como o fortalecimento dos pequenos mercados em áreas de vulnerabilidade, são estratégias viáveis, visto que são estabelecimentos amplamente utilizados pela população vulnerabilizada. No entanto, é preciso destacar que essas estratégias podem não ser suficientes para a construção de um ambiente alimentar de varejo saudável em áreas urbanas, especialmente em grandes metrópoles.

No âmbito municipal, entretanto, o cenário regulatório do Rio de Janeiro ainda se mostra insuficiente para sustentar essa agenda de forma integrada. A *Lei nº 6.412/2018* ^57^, que instituiu a Política Municipal de Segurança Alimentar e Nutricional (PMSAN), estabeleceu um marco institucional relevante, mas sua implementação carece de instrumentos que incidam de forma efetiva sobre o varejo alimentar. Da mesma forma, normas como o *Decreto nº 45.585/2018* (feiras livres) ^58^ e a *Lei nº 5.333/2011* ^59^ tratam, sobretudo, de aspectos operacionais e sanitários, sem incorporar diretrizes que considerem a saudabilidade, a equidade territorial ou o ordenamento urbano voltado ao acesso a alimentos *in natura*.

Tais normas evidenciam que o ambiente alimentar carioca segue estruturado majoritariamente pelas dinâmicas de mercado com baixa intervenção estatal. Assim, embora estratégias como o fortalecimento de pequenos mercados e a ampliação de equipamentos que priorizem a disponibilidade de alimentos frescos sejam oportunas, dependem de maior integração com o planejamento urbano municipal, incluindo mecanismos de zoneamento e regulação da oferta de ultraprocessados.

É fundamental ressaltar que a maior utilização de locais de venda de alimentos *in natura* e minimamente processados por grupos de maior vulnerabilidade socioeconômica, embora represente um padrão protetor, não pode ser interpretada como uma escolha plena e sustentável. Este perfil é frequentemente moldado por restrições orçamentárias e pela proximidade geográfica do pequeno varejo, e não por uma condição de dignidade plena. Portanto, a dignidade inerente ao avanço socioeconômico por meio de políticas de distribuição de renda, acesso à educação e ao mercado de trabalho também deve ser priorizada. Entretanto, é um imperativo de política pública que o esforço para garantir essa melhoria de renda seja acompanhado pela regulamentação e pelo fortalecimento do ambiente alimentar. Sem essa intervenção protetora, o aumento da capacidade de compra dessas famílias pode ser capturado pela ampla e agressiva oferta de produtos ultraprocessados no grande varejo. Desta forma, a falta de um ambiente alimentar adequado impediria que o ganho de renda se convertesse em benefícios reais para a saúde, podendo anular o padrão de acesso mais favorável observado neste manuscrito.

Apesar da baixa frequência de aquisição de alimentos no segmento informal, este se mostrou presente no ambiente alimentar do Município do Rio de Janeiro. É preciso reconhecer que esse tipo de comércio é importante na dinâmica de acesso aos alimentos nos grandes centros urbanos, mas para entender a dinâmica do funcionamento e da relação comerciante/consumidor é necessária a construção de estudos com objetivos e metodologias específicas para que se entenda a importância desse componente no ambiente alimentar.

Contudo, o presente estudo apresentou algumas limitações. As informações utilizadas foram baseadas em autorrelato, o que implica a ausência de um registro objetivo das compras. A não utilização de uma caderneta de compras, instrumento que permite registrar de forma contínua e detalhada os itens adquiridos, decorreu do desenho do estudo original, o qual permitiu detalhar o acesso aos equipamentos de venda de alimentos a partir de uma lista de itens previamente definida com os principais alimentos adquiridos e consumidos, conforme a POF 2017/2018 ^29^. Reconhece-se também que o estudo não contemplou o ambiente alimentar virtual, como os aplicativos de entrega, que vêm assumindo influência crescente na aquisição de alimentos. Adicionalmente, a estratégia de análise multivariada, ao ajustar simultaneamente variáveis socioeconômicas que compõem um mesmo processo de estratificação social, pode ter subestimado o efeito total e cumulativo das desigualdades. Porém, por se tratar de um estudo exploratório, apresentar os resultados mutuamente ajustados contribui para o entendimento das múltiplas relações entre os indicadores socioeconômicos considerados e as escolhas dos locais de aquisição de alimentos no território avaliado. Esse tipo de análise permite apresentar valores de chance em diferentes direções para as associações testadas e assim nortear gestores e atores sociais que atuam na formulação de políticas públicas voltadas aos temas estudados neste manuscrito.

## Conclusão

Este estudo demonstrou que os pequenos mercados e os locais de venda de alimentos *in natura* e minimamente processados configuram importantes equipamentos de compra de alimentos por famílias marcadas por desigualdades sociais no Município do Rio de Janeiro.

Tais achados demonstram a necessidade de uma atuação efetiva do Estado por meio da implementação de políticas públicas que incentivem a expansão do número de feiras e mercados populares, bem como o fortalecimento do pequeno varejo local.

Adicionalmente, é fundamental que estratégias voltadas à diminuição das desigualdades sociais sejam inseridas nas ações do campo da alimentação e nutrição, de modo a diversificar o ambiente alimentar e assegurar o acesso equitativo a alimentos saudáveis para toda a população.

Embora os supermercados tenham sido identificados como o principal local de aquisição de alimentos pelas famílias cariocas, este cenário aponta para a urgência de mecanismos que regulem sua velocidade de expansão. O crescimento desregulado do grande varejo reduz a capacidade de concorrência de locais essenciais como feiras e o pequeno varejo tradicional, além de favorecer o consumo de produtos ultraprocessados pela população devido à sua ampla e ostensiva oferta.

Desta forma, o fortalecimento do ambiente alimentar saudável, por meio da proteção e expansão de feiras e do varejo tradicional no entorno das residências das famílias, não apenas garantirá o direito humano à alimentação adequada por meio do acesso a alimentos saudáveis e com preço justo, mas também impactará positivamente na garantia de outros direitos fundamentais, permitindo a plena realização do conceito de segurança alimentar e nutricional.

## Data Availability

Não se aplica.
